# Evaluation of mechanical ventilation modes in the laparoscopic perioperative period with electrical impedance tomography

**DOI:** 10.1371/journal.pone.0331194

**Published:** 2025-09-08

**Authors:** Zhiwei Li, Yang Wu, Yao Yu, Kai Liu, Hang Tian, Jiafeng Yao, Qiuju Cheng

**Affiliations:** 1 College of Mechanical and Electrical Engineering, Nanjing University of Aeronautics and Astronautics, Nanjing, China; 2 College of Mechanical and Electrical Engineering, Nanjing Forestry University, Nanjing, China; 3 Department of Anesthesiology, Guangzhou Women and Children’s Medical Center, Guangzhou Medical University, Guangdong Provincial Clinical Research Center for Child Health, Guangzhou, China; 4 College of Physics & Optoelectronic Engineering, Jinan University, Guangzhou, China; Maulana Azad Medical College, INDIA

## Abstract

**Purpose:**

Uncertainty persists regarding the optimal mode of mechanical ventilation for laparoscopic perioperative periods. Electrical impedance tomography (EIT) is an effective tool for monitoring and guiding lung-protective ventilation. This study aimed to compare the effects of pressure-controlled ventilation-volume guaranteed (PCV-VG) and volume-controlled ventilation (VCV) on pulmonary ventilation during laparoscopic surgery.

**Method:**

This trial was a randomized crossover study, with the laparoscopic perioperative period divided into five phases: pre-anesthesia induction (AWAKE), post-anesthesia induction (BEGIN), the first phase of surgery (MIDDLE-1), the second phase of surgery (MIDDLE-2), and pre-wakefulness (END). EIT data were recorded at each phase, and EIT parameters were calculated to quantify pulmonary ventilation performance in both spatial and temporal dimensions.

**Results:**

During the surgical period, plateau pressure (Pplat) and driving pressure (ΔP) under PCV-VG were lower, while the oxygenation index (PaO_2_/FiO_2_) was higher compared to VCV. Furthermore, PCV-VG was associated with an increased center of ventilation (45.1 ± 3.7 vs. 41.7 ± 3.5, P < 0.05), a decrease in global inhomogeneity (GI) (0.85 ± 0.16 vs. 1.06 ± 0.30, P < 0.05), and a reduction in regional ventilation delay index (RVDI) (10.5 ± 4.5 vs. 15.9 ± 5.3, P < 0.05), when compared to VCV. The improvements in pulmonary ventilation were more pronounced with PCV-VG compared to VCV during the surgical period, compared to the non-surgical period.

**Conclusion:**

EIT parameters reveal significant differences in ventilation between VCV and PCV-VG during the laparoscopic perioperative period. PCV-VG improves ventilation inhomogeneity and mitigates ventilation delay caused by the Trendelenburg position and pneumoperitoneum during surgery. PCV-VG is more effective than VCV in optimizing intraoperative lung ventilation.

**Trial registration:**

ClinicalTrials.gov ChiCTR2400089365

## Introduction

Perioperative lung-protective ventilation reduces the risk of postoperative pulmonary complications and improves lung function and prognosis [1–3]. Lung-protective ventilation strategies aim to minimize intraoperative lung injury by optimizing mechanical ventilation parameters [4–6].

During the perioperative period of laparoscopic surgery, changes in position and pneumoperitoneum contribute to the development of pulmonary atelectasis, increasing the risk of localized lung injury [7,8]. Timely adaptation of lung-protective ventilation strategies is essential in this period. Respiratory compliance and the oxygenation index (PaO_2_/FiO_2_) are key parameters for monitoring lung function intraoperatively. However, evaluating ventilatory status using only these parameters has limitations [9,10].

Electrical impedance tomography (EIT) is a non-invasive, dynamic, and visual medical monitoring technique that has proven valuable in guiding lung-protective ventilation strategies [11–14]. EIT provides real-time information on impedance changes in the lungs and allows the ventilation distribution to be visualized in reconstructed images. In clinical anesthesia, EIT is primarily used to guide prone therapy, monitor lung tidal volume, and detect pleural effusions [15–17]. Notably, EIT has demonstrated significant advantages in identifying optimal positive end-expiratory pressure (PEEP) and reducing localized lung collapse [18–20]. However, uncertainty remains regarding the most effective mode of mechanical ventilation for lung-protective strategies during the laparoscopic perioperative period, with clinical experience and subjectivity largely guiding decision-making.

Pressure-controlled ventilation-volume guaranteed (PCV-VG) and volume-controlled ventilation (VCV) are two commonly used modes of mechanical ventilation in anesthesia. In VCV, the anesthesia machine delivers the preset tidal volume (TV) with a constant flow at a preset respiratory rate during a fixed inspiratory time. PCV-VG is designed to ensure both stable ventilation pressure and a preset tidal volume. In PCV-VG, the anesthetist adjusts the pressure of each breath to achieve the target tidal volume, based on real-time measurements of lung compliance and resistance. While most studies have evaluated these two ventilation modes in terms of respiratory mechanics, they still have limitations [21,22].

In this study, we evaluated the effects of PCV-VG and VCV on lung ventilation during the perioperative laparoscopic period from both spatial and temporal perspectives, based on EIT ventilation parameters. We hypothesized that PCV-VG would be more effective than VCV during the laparoscopic perioperative period.

## Materials and methods

### Experimental ethics

This study adopted a randomized crossover design, in which patients received two ventilation modes in a randomly assigned sequence to control for potential order effects. The trial was approved by the Ethics Committee of Guangzhou Women and Children Medical Centre (Guangzhou, China; ID: Sui Women’s and Children’s Corun Tong Zi [2022] No. 292B00). The Consolidated Standards of Reporting Trials (CONSORT) guideline was followed for reporting (S1 File). This trial was conducted at the Guangzhou Women and Children’s Medical Center, beginning on January 1, 2024, and ending on December 1, 2024. The trial was registered in ChiCTR2400089365 after participant recruitment began due to an administrative oversight during the initial phase of the study. Although the clinical trial registration (ChiCTR2400089365) initially recorded the study as a retrospective design focusing on PPCs, the final protocol implemented a prospective randomized crossover trial evaluating EIT ventilation parameters. The discrepancy arose due to administrative delays in trial registration. The Authors confirm that all ongoing and related trials for this drug/intervention are registered. All participants were verbally informed and signed informed consent before enrollment.

### Inclusion and exclusion criteria

**Fig 1:**
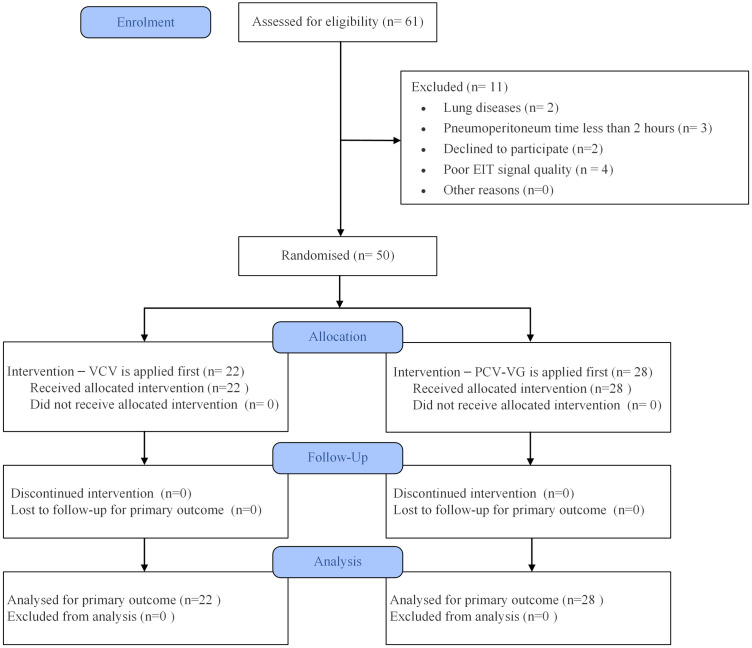
CONSORT flow chart of included patients.

This study was exploratory in nature, and no formal a priori power calculation was performed. However, a post hoc sample size estimation indicated that approximately 54 participants would be required to ensure adequate statistical power (S3 File). A total of 61 cardiorespiratory-healthy female patients requiring laparoscopic surgery were included (as shown in Fig 1). The surgeries were primarily performed for benign ovarian tumors, uterine fibroids, and secondary infertility. Inclusion criteria: (1) Age 18-59 years old. (2) American Society of Anesthesiologists (ASA) classification I-II. (3) Body mass index (BMI) 18.5–28 kg/m^2^. Exclusion criteria: (1) Patients with chronic lung diseases, infections, or other serious pulmonary complications, such as acute respiratory failure. (2) Patients with a history of thoracic or pulmonary surgery, or those with alveolar disorders. (3) Patients with severe cardiovascular, cerebrovascular, hepatic, renal, or neurological diseases affecting respiratory function in the preoperative period. (4) Patients who have smoked within 8 weeks before surgery. (5) Surgical time less than 1 hour or greater than 6 hours, or pneumoperitoneum time less than 2 hours. (6) Intraoperative conversion to open surgery. (7) Poor EIT signal quality.

Participants were randomly assigned to receive either VCV or PCV-VG ventilation modes in a randomized crossover design. The allocation sequence was generated using a computer-based random number generator. The randomization was performed by an independent research assistant who was not involved in patient treatment or data analysis, maintaining the objectivity of the study. Additionally, the investigator responsible for the outcome assessments was blinded to the randomization sequence, ensuring that the analysis was free from potential bias.

### Study protocol

**Fig 2:**
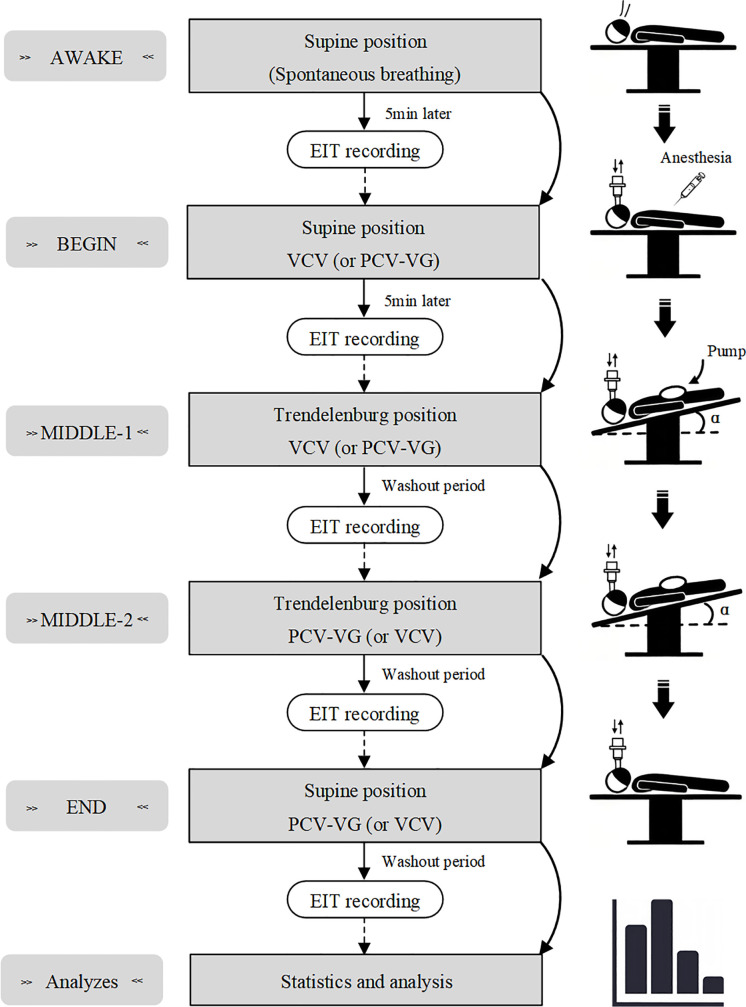
Flowchart of the perioperative period and the study. EIT, electrical impedance tomography; AWAKE = before the induction of anesthesia; BEGIN = the beginning of anesthesia induction and VCV (or PCV-VG); MIDDLE-1 = the first phase of the surgery and VCV (or PCV-VG); MIDDLE-2 = the second phase of the surgery and PCV-VG (or VCV); END = before postoperative wakefulness and PCV-VG (or VCV); Pump, pneumoperitoneum operation. The tilt angle of the operating table is α = 20°.

Fig 2 shows the study protocol. The laparoscopic perioperative period was divided into five phases. In the AWAKE phase, patients were supine, awake, and spontaneously breathing. The BEGIN phase followed anesthesia induction, without alveolar recruitment maneuvers. Patients were supine and received mechanical ventilation (VCV or PCV-VG). The surgical period then began. In the MIDDLE-1 phase, patients were placed in the Trendelenburg position with pneumoperitoneum, and the ventilation mode was maintained. In the MIDDLE-2 phase, the ventilation mode was changed. In the END phase, patients were released from the pneumoperitoneum in the supine position and transferred to the resuscitation room with the ventilation mode maintained. Patients were connected to a monitoring system in the operating theatre to track heart rate, blood pressure, and oxygen saturation. To more sensitively monitor impedance signals, the EIT electrode belt was placed slightly below the breast, between the 4th and 5th ribs [23]. Anesthesia was induced with remimazolam (0.3 mg/kg), sufentanil (0.3 *μ*g/kg), cisatracurium (0.3 mg/kg), and propofol (2 mg/kg).

Mechanical ventilation was set with a PEEP of 5 cmH_2_O, an initial tidal volume of 7 ml/kg, an I:E ratio of 1:2, and a respiratory rate (RR) adjusted to maintain a partial pressure of end-tidal carbon dioxide (P_ET_CO_2_) of 35 ± 5 mmHg. FiO_2_ was maintained at 30%−50%, and intra-abdominal pressure was uniformly set at 12 mmHg.

EIT data were collected using the EIT-1000 (Suzhou Jiantong Medical Technology Co., Ltd., Jiangsu, China). The output signal of the EIT system was a 1mA, 122kHz safety current. The EIT belt consisted of 16 electrodes, and the skin around the chest was cleaned with saline before electrode placement to minimize the effects of contact impedance. Impedance tomography images were recorded at 20 fps with stable and adequate electrode contact.

### Experimental program

A washout period was incorporated to minimize the cumulative effect of the previous phase on the subsequent one. The washout period was defined as the first 30 minutes following the transition to the next phase and was applied to the MIDDLE-1, MIDDLE-2, and END phases. Existing clinical studies indicate that a 30-minute washout period is sufficient for respiratory mechanics, airway pressure, and oxygenation parameters to return to baseline after a change in ventilation mode [24,25]. After the washout period, EIT data were recorded for 5 minutes. The image reconstruction algorithm for EIT used Gaussian-Newton regularization, with image smoothness enhanced through convolutional filtering [26]. The pixel size of each image was 128 × 128.

### Experimental parameters

**Fig 3:**
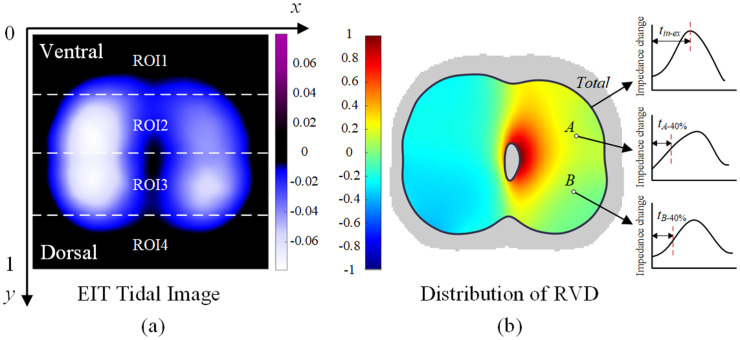
Schematic diagram of EIT. (a) EIT tidal image. The EIT tidal image is divided equally from ventral to dorsal into four regions, denoted ROI 1-ROI 4. Using the ventral line as the reference axis, the y-axis is normalized to 0 for the ventral and 1 for the dorsal. (b) RVDI distribution plot. Each pixel point in the RVDI plot represents the time required to reach 40% of the inspiratory maximum.

Center of ventilation (CoV), global inhomogeneity (GI), and regional ventilation delay index (RVDI) are commonly used parameters to assess lung function in EIT [27–29]. The EIT tidal image is generated by calculating impedance changes between the end-inspiratory and end-expiratory moments of a single respiratory cycle, as shown in Fig 3. Each pixel in the image represents relative impedance changes across the chest. EIT parameters are derived from these tidal images. A CoV value greater than 50 indicates a larger proportion of dorsal ventilation. In comparison, a lower value suggests the dominance of ventral ventilation, depending on the direction of the prescribed y-axis. GI reflects the homogeneity of pulmonary ventilation, with lower GI values indicating more uniform ventilation. RVDI quantifies the delay in pulmonary ventilation, where lower values signify less variation in ventilation timing across different lung regions.

For each patient, the EIT tidal images for the five phases were standardized to the same color scale range, and EIT ventilation parameters were calculated within the same contour. The averages of the EIT parameters from five consecutive respiratory cycles were computed for each phase.

### Statistical analysis

The experimental subjects in this study were female patients requiring laparoscopic surgery. The mechanical ventilation phases during the laparoscopic perioperative period were divided into non-surgical and surgical periods. The non-surgical period includes the BEGIN and END phases, while the surgical period includes the MIDDLE-1 and MIDDLE-2 phases.

This experiment was a randomized crossover statistical study of clinical parameters across different phases during the perioperative laparoscopic period. The statistical parameters included heart rate (HR), mean arterial pressure (MAP), arterial partial pressure of carbon dioxide (PaCO_2_), pulmonary dynamic compliance (Cdyn), peak pressure (Ppeak), plateau pressure (Pplat), driving pressure (ΔP), and oxygenation index (PaO_2_/FiO_2_). All clinical indicators were recorded during the EIT data collection period. The primary outcomes were changes in EIT ventilation images (CoV, GI and RVDI). The secondary outcomes included conventional respiratory parameters and clinical variables such as Ppeak, Pplat, ΔP, Cdyn, PaO₂/FiO₂, MAP, HR, and PaCO₂.

The normality of continuous variables was assessed using the Shapiro-Wilk test. Data that satisfied normality are presented as mean ± SD, with comparisons between phases conducted using paired t-tests. For continuous variables that did not meet the assumption of normality, data are presented as medians (interquartile ranges), and comparisons were made using Wilcoxon tests. Statistical significance was defined as P < 0.05. All statistical analyses were performed using SPSS version 26.0.

## Results

**Fig 4:**
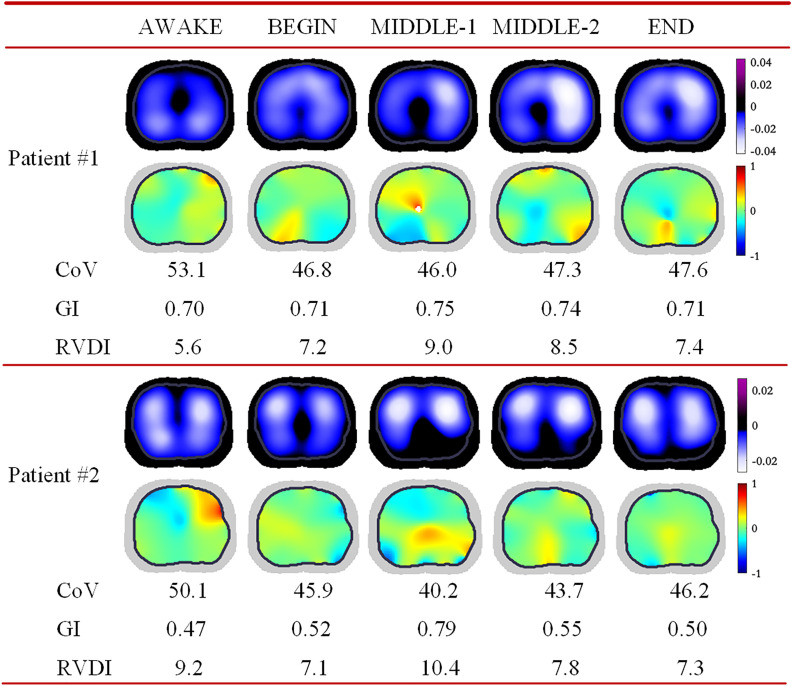
Samples of EIT tidal images and RVDI distribution plots. The sequence of mechanical ventilation for patient #1 and patient #2 was VCV to PCV-VG and PCV-VG to VCV, respectively. AWAKE = before the induction of anesthesia; BEGIN = the beginning of anesthesia induction and VCV (or PCV-VG); MIDDLE-1 = the first phase of the surgery and VCV (or PCV-VG); MIDDLE-2 = the second phase of the surgery and PCV-VG (or VCV); END = before postoperative wakefulness and PCV-VG (or VCV); CoV = center of ventilation. GI = global inhomogeneity. RVDI = regional ventilation delay index.

A total of 50 patients were statistically analyzed. Patient characteristics are shown in Table 1. Two representative samples of EIT tidal images and RVDI distribution from the perioperative period are shown in Fig 4.

**Table 1:** Patient characteristics.

Basic Parameters	Laparoscopic surgery (N = 50)
Age, median (IQR), year	41 (27 - 52)
Height, mean ± SD, cm	155.2 ± 5.6
Weight, mean ± SD, kg	57.7 ± 14.5
BMI, mean ± SD, kg/m²	22.3 ± 4.2
Duration of surgery, mean ± SD, min	154.8 ± 69.4
Respiratory rate, median (IQR), per min	14 (10 - 16)

The data are presented as the mean ± SD or median (IQR).

Table 2 shows a comparison of relevant ventilation parameters between VCV and PCV-VG. In the non-surgical period, PCV-VG was associated with a decrease in ΔP compared to VCV. During the surgical period, differences in Pplat, ΔP, and PaO_2_/FiO_2_ were observed between the two modes. No notable differences were observed for the remaining parameters (P > 0.05).

**Table 2:** Ventilation parameters.

	Laparoscopic surgery (N = 50)			
	VCV	PCV-VG	*P* Value	Cohen’s d (r) Value
HR (per min)				
Non-surgical period	68.6 ± 7.5	69.7 ± 6.3	0.405*	0.22
Surgical period	70.4 ± 5.9	72.2 ± 7.1	0.327*	0.22
*P* Value	0.281*	0.644*	/	/
Cohen’s d (r) Value	0.21	0.15	/	/
MAP (mmHg)				
Non-surgical period	75.8 ± 2.9	77.4 ± 3.0	0.523*	0.25
Surgical period	83.2 ± 2.7	85.4 ± 3.2	0.691*	0.02
*P* Value	<0.001*	0.003*	/	/
Cohen’s d (r) Value	**0.87**	**0.77**	/	/
PaCO_2_ (mmHg)				
Non-surgical period	40.7 ± 1.5	43.0 ± 1.6	0.070*	0.24
Surgical period	44.2 ± 1.4	46.5 ± 1.8	0.064*	0.32
*P* Value	0.002*	0.021*	/	/
Cohen’s d (r) Value	**1.21**	**0.74**	/	/
Cdyn (mL/cmH_2_O)				
Non-surgical period	37.4 ± 2.5	39.2 ± 2.7	0.224*	0.52
Surgical period	25.3 ± 1.4	27.0 ± 1.8	0.181*	0.49
*P* Value	<0.001*	<0.001*	/	/
Cohen’s d (r) Value	**1.93**	**1.45**	/	/
Ppeak (cmH_2_O)				
Non-surgical period	14 (11 – 15)	14 (11 – 16)	0.501^₭^	0.25
Surgical period	21 (15 – 28)	20 (14 – 26)	0.053^₭^	0.46
*P* Value	**<0.001** ^₭^	**0.002** ^₭^	/	/
Cohen’s d (r) Value	**0.88**	**0.71**	/	/
Pplat (cmH_2_O)				
Non-surgical period	8 (6 – 10)	7 (5 – 9)	0.082^₭^	0.35
Surgical period	13 (8 – 17)	11 (7 – 14)	**0.024** ^₭^	**0.57**
*P* Value	**<0.001** ^₭^	**<0.001** ^₭^	/	/
Cohen’s d (r) Value	**0.88**	**0.81**	/	/
ΔP (cmH_2_O)				
Non-surgical period	5 (2 – 8)	4 (2 – 6)	**0.031** ^₭^	**0.49**
Surgical period	10 (5 – 15)	8 (5 – 10)	**0.028** ^₭^	**0.59**
*P* Value	**<0.001** ^₭^	**<0.001** ^₭^	/	**/**
Cohen’s d (r) Value	**0.88**	**0.82**	**/**	**/**
PaO_2_/FiO_2_ (mmHg)				
Non-surgical period	485 (431 - 545)	490 (440 - 553)	0.288^₭^	0.16
Surgical period	453 (414 - 516)	475 (425 - 542)	**0.039** ^₭^	**0.48**
*P* Value	**0.003** ^₭^	**0.040** ^₭^	/	/
Cohen’s d (r) Value	**0.63**	**0.47**	/	/

Measurements were taken at ventilation modes of VCV or PCV-VG. The data are presented as the mean ± SD or median (IQR). Each group of data corresponds to its row and column. The non-surgical period includes BEGIN and END. The surgical period includes MIDDLE-1 and MIDDLE-2. *P* < 0.05 is shown in bold. * is the paired t-test. ₭ is the Wilcoxon test. Cohen’s d for the paired t-test and r for the Wilcoxon test.

**Fig 5:**
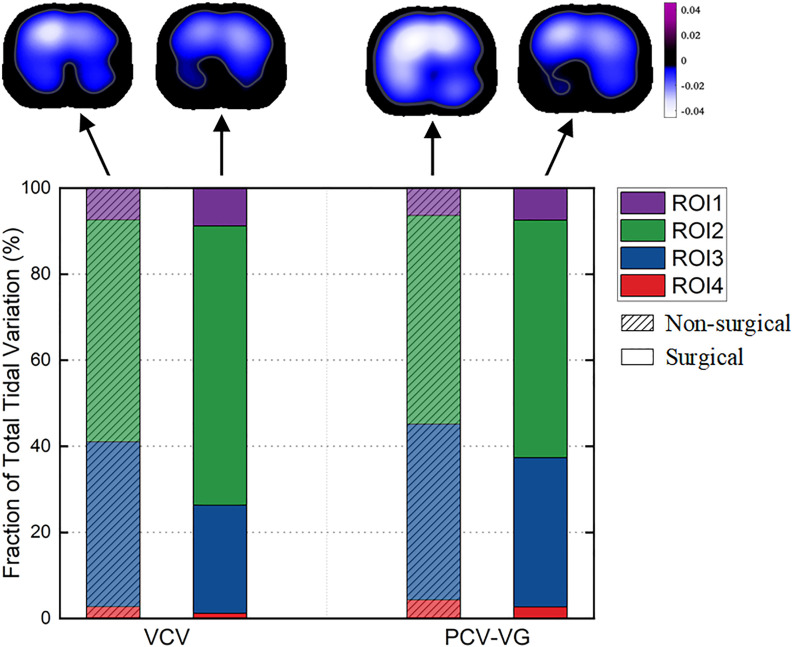
Percentage of total tidal variation per region of interest. Each stacked bar shows the ventilation distribution in the four regions of interest (ROI 1-ROI 4; also see Table 3).

Fig 5 shows the percentage of total tidal variation per region of interest (ROI). Most of the ventilation is concentrated in ROI 2 and ROI 3. In the non-surgical period, PCV-VG showed a significant decrease in the ventilation percentage of ROI 2 by 7% compared to VCV, and an 8% decrease in the surgical period (P < 0.05). During the surgical period, the ventilation percentage of ROI 2 appeared to increase by 10% in VCV and 9% in PCV-VG relative to the non-surgical period.

**Table 3:** Parameters of EIT.

	Laparoscopic surgery (N = 50)			
	VCV	PCV-VG	*P* Value	Cohen’s d (r) Value
ROI 1				
Non-surgical period	8 (6 – 10)	7 (5 – 8)	**0.024** ^₭^	**0.50**
Surgical period	9 (7 – 13)	8 (5 – 12)	**0.021** ^₭^	**0.51**
*P* Value	0.118^₭^	**0.025** ^₭^	/	/
Cohen’s d (r) Value	0.35	**0.50**	/	/
ROI 2				
Non-surgical period	55 (48 - 61)	48 (42 – 55)	**0.026** ^₭^	**0.50**
Surgical period	65 (62 - 69)	57 (52 - 65)	**<0.001** ^₭^	**0.87**
*P* Value	**<0.001** ^₭^	**0.001** ^₭^	/	**/**
Cohen’s d (r) Value	**0.88**	**0.77**	**/**	**/**
ROI 3				
Non-surgical period	35 (28 – 44)	42 (38 – 47)	0.061	0.42
Surgical period	25 (16 - 35)	33 (25 - 42)	**<0.001** ^**₭**^	**0.88**
*P* Value	**<0.001** ^**₭**^	**0.001** ^**₭**^	/	/
Cohen’s d (r) Value	**0.88**	**0.74**	/	/
ROI 4				
Non-surgical period	3 (2 – 4)	4 (3 – 6)	**0.002** ^**₭**^	**0.71**
Surgical period	2 (0 - 3)	3 (1 – 5)	**0.003** ^**₭**^	**0.66**
*P* Value	**0.011** ^**₭**^	**0.004** ^**₭**^	**/**	**/**
Cohen’s d (r) Value	**0.57**	**0.64**	**/**	**/**
CoV				
Non-surgical period	45.6 ± 4.8	49.3 ± 3.0	0.003*	**0.75**
Surgical period	41.7 ± 3.5	45.1 ± 3.7	<0.001*	**1.82**
*P* Value	<0.001*	<0.001*	/	**/**
Cohen’s d (r) Value	**1.42**	**1.02**	**/**	**/**
GI				
Non-surgical period	0.82 ± 0.14	0.73 ± 0.11	0.149	0.34
Surgical period	1.06 ± 0.30	0.85 ± 0.16	0.002*	**0.81**
*P* Value	<0.001*	0.002*	**/**	/
Cohen’s d (r) Value	**1.02**	**0.82**	/	/
RVDI				
Non-surgical period	10.8 ± 4.3	7.8 ± 3.6	0.001*	**0.84**
Surgical period	15.9 ± 5.3	10.5 ± 4.5	<0.001*	**1.19**
*P* Value	<0.001*	<0.001*	/	
Cohen’s d (r) Value	**1.51**	**1.09**		

The data are presented as the mean ± SD or median (IQR). Each group of data corresponds to its row and column. ROI 1–ROI 4 are presented as median (IQR). CoV, GI and RVDI are presented as the mean ± SD. Region of interest ROI 1 is ventral, whereas ROI 4 is dorsal. Pump, pneumoperitoneum operation. The non-surgical period includes BEGIN and END. The surgical period includes MIDDLE-1 and MIDDLE-2. *P* < 0.05 is shown in bold. * is the paired t-test. ₭ is the Wilcoxon test. Cohen’s d for the paired t-test and r for the Wilcoxon test.

**Fig 6:**
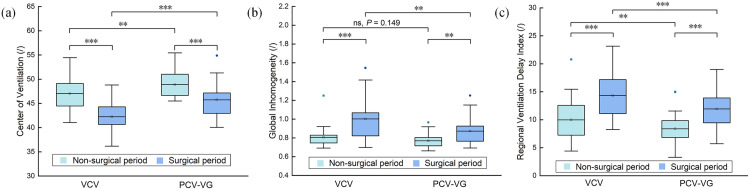
Box plot (median with 25th and 75th percentiles) of EIT parameters in VCV and PCV-VG. (a) Center of ventilation (CoV). (b) Global inhomogeneity (GI). (cl) Regional ventilation delay index (RVDI). ns, no significant difference, * P < 0.05, ** P < 0.01, *** P < 0.001.

Table 3 shows a comparison of EIT parameters between VCV and PCV-VG. Fig 6 shows box plots of these parameters. In the non-surgical period, PCV-VG was associated with an increase in CoV (mean difference: 3.7) and a decrease in RVDI (mean difference: 3) compared to VCV. In the surgical period, CoV was higher by 3.4 units, while GI and RVDI showed reductions of 0.21 and 5.4 with PCV-VG, respectively. These differences were statistically significant (P < 0.05).

In VCV, CoV showed a decrease of 3.9, accompanied by increases of 0.24 in GI and 5.1 in RVDI in the surgical period compared to the non-surgical period (P < 0.05). In PCV-VG, CoV also showed a decrease of 4.2, while GI and RVDI decreased by 0.12 and 2.7 in the surgical period compared to the non-surgical period (P < 0.05). While these comparisons reached statistical significance (P < 0.05), the findings should be interpreted with caution due to the exploratory nature of the study.

## Discussion

This trial compared the impacts of VCV and PCV-VG on pulmonary ventilation during the laparoscopic perioperative period. Lung ventilation was quantified using EIT image parameters. The main findings were: (1) In both the non-surgical and surgical periods, pulmonary ventilation with PCV-VG appeared to be more evenly distributed, with trends toward improved global homogeneity and reduced ventilation delay compared to VCV. (2) In the surgical period, the ventilation region in the EIT image shifted ventrally, with decreased global homogeneity and increased ventilation delay compared to the non-surgical period.

When the patients were mechanically ventilated after induction of anesthesia, CoV was mostly below 50, indicating that ventilation was concentrated in the ventral region. This clinical observation is consistent with previous studies [30]. During spontaneous breathing in the supine position, gravity causes ventilation to shift dorsally [31]. However, mechanical ventilation during general anesthesia prevents the dorsal alveoli from fully opening, concentrating ventilation in the ventral region. Applying high PEEP can effectively improve dorsal lung atelectasis [32]. This study used low PEEP to enhance the discernible effect between the ventilation modes, in line with previous research [33]. No hypoxemia was observed during the experimental follow-up of low PEEP.

Previous studies have shown that PCV-VG significantly lowers peak inspiratory pressure and increases dynamic compliance compared to VCV [34]. Toker et al. reported that PCV-VG improved oxygenation and ventilation parameters during laparoscopic gynecologic surgery [35]. In our study, PCV-VG enhanced the uniformity of pulmonary ventilation distribution and reduced ventilation delay in both the non-surgical and surgical periods compared to VCV. Additionally, a decreasing trend in Pplat and ΔP was observed. Consistently, Jung Min Lee et al. reported that PCV-VG resulted in smaller changes in airway pressure compared to VCV during robot-assisted laparoscopic gynecologic surgery in the Trendelenburg position [34]. This phenomenon can be explained by the fact that PCV-VG reduces airway resistance and improves respiratory compliance [36]. Furthermore, it helps reduce alveolar collapse, ensuring more effective ventilation. Notably, the variability in pulmonary ventilation parameters between the two ventilation modes was relatively small in our study, likely due to the inclusion of patients who were cardiopulmonary healthy. However, the variability in lung ventilation would likely be more pronounced in patients with alveolar disease or those undergoing one-lung ventilation.

When comparing the differences in mechanical ventilation modes between the non-surgical and surgical periods, we observed that pulmonary ventilation improvements were more evident with PCV-VG than with VCV during the surgical period. Specifically, PaO_2_/FiO_2_ showed a more substantial improvement during surgery with PCV-VG. In contrast, no significant difference in global inhomogeneity (GI) was observed between VCV and PCV-VG during the non-surgical period. This suggests that mechanical ventilation modes may have a lesser impact on pulmonary ventilation during the non-surgical period. On the other hand, PCV-VG may help alleviate poor ventilation associated with the Trendelenburg position with pneumoperitoneum during the surgical period.

Additionally, the region of lung ventilation during the surgical period was shifted ventrally compared to the non-surgical period, which is consistent with previous studies [37]. The observed decrease in PaO_2_/FiO_2_ indicates a deterioration in alveolar ventilatory exchange function. We further found that changes in position, as reflected by the GI and RVDI parameters, lead to worsened ventilation. Physiologically, the Trendelenburg position with pneumoperitoneum forces the diaphragm upward, increasing the compression on the dorsal side of the lungs and resulting in dorsal alveolar collapse. Moreover, we found that ΔP increased and respiratory compliance decreased, likely due to increased airway resistance caused by the compression, which subsequently reduces pulmonary ventilation.

A randomized crossover study design was employed, using the same patient to account for individual variability in pulmonary ventilation. The unique aspect of our study lies in the comparison of VCV and PCV-VG on pulmonary ventilation, incorporating EIT ventilation parameters alongside other clinical indices, such as PaO_2_/FiO_2_, Cdyn, and airway pressure.

In this study, to minimize residual effects from mode transitions, a 30-minute washout period was implemented before the initiation of the MIDDLE-1, MIDDLE-2, and END phases. This duration was based on previous crossover ventilation studies. Ceylan et al. demonstrated that respiratory parameters stabilize within approximately 30 minutes after a change in ventilation mode in pediatric patients [24]. Bonacina et al. reported similar recovery times in postoperative infants under different ventilatory supports [25]. Existing clinical studies indicate that a 30-minute interval is sufficient for respiratory mechanics, airway pressure, and oxygenation parameters to return to baseline after a change in ventilation mode. This recovery window allows for the clearance of transient physiological effects induced by the preceding ventilation mode, thereby ensuring that subsequent measurements more accurately reflect the effects of the new mode rather than residual influences. While longer washout periods could theoretically further reduce carryover, they may unnecessarily prolong the surgical procedure and introduce additional variability unrelated to ventilation mode. Therefore, the 30-minute period used in this study represents a practical balance between physiological recovery and procedural feasibility.

The limitations of this study include: (1) The study population was limited to cardiorespiratory-healthy female patients undergoing laparoscopic surgery, which may restrict the generalizability of the findings. Larger and more diverse samples, including patients with obesity, lung disease, and other comorbidities, are needed in future studies to validate these findings and enhance their applicability across different patient populations. (2) The potential cumulative effect of anesthesia duration and mechanical ventilation, which may influence the independence of each phase. To minimize this effect, we employed a randomized ventilation sequence and incorporated a washout period in our experimental protocol. (3) Given the exploratory design and absence of a priori power calculation, the reported P values should be interpreted conservatively. Rather, they are hypothesis-generating and highlight trends that warrant further investigation in larger, well-powered studies.

## Conclusions

Significant differences in EIT ventilation parameters were observed between VCV and PCV-VG during the laparoscopic perioperative period. Compared to VCV, PCV-VG improves ventilation inhomogeneity and reduces ventilation delay, particularly due to the Trendelenburg position with pneumoperitoneum. Using PCV-VG during the surgical period enhances PaO_2_/FiO_2_, reduces airway resistance, and proves more beneficial for pulmonary ventilation.

## Supporting information

S1 File CONSORT_checklist. (DOCX)

S2 File Trial protocol. (DOCX)

S3 File Power calculation. (DOCX)
