# Supplementary material for: Evaluation of mechanical ventilation modes in the laparoscopic perioperative period with electrical impedance tomography
Source: PLoS One. 2025 Sep 8;20(9):e0331194. doi: 10.1371/journal.pone.0331194 (PMC12416645; doi:10.1371/journal.pone.0331194)
Supplement: S2 File — (DOCX) [file pone.0331194.s002.docx]

**Study Protocol**

1. **Study Population**

The study will include female patients diagnosed with benign ovarian tumors, uterine fibroids, or secondary infertility who are scheduled for laparoscopic diagnosis and treatment. Inclusion criteria: (1) Age between 18 and 59 years; (2) American Society of Anesthesiologists (ASA) physical status I–II; (3) Body Mass Index (BMI) between 18.5–28 kg/m². Exclusion criteria: (1) Patients with chronic pulmonary diseases, infections, or severe pulmonary complications such as acute respiratory failure; (2) History of thoracic or pulmonary surgery or alveolar disorders; (3) Severe cardiovascular, cerebrovascular, hepatic, renal, or neurological diseases affecting respiratory function; (4) Smoking history within 8 weeks prior to surgery. Elimination criteria: (1) Surgical time less than 1 hour or longer than 6 hours, or pneumoperitoneum time less than 2 hours; (2) Intraoperative conversion to open surgery.

1. **Sample Size**

This study adopts a randomized crossover design with a target enrollment of 50 patients. After accounting for signal loss and data interference during surgery, the final number of patients with valid EIT data is 50. No formal sample size calculation was performed; the study is exploratory in nature.

1. **Group Allocation and Experimental Process**

A randomized crossover design will be used. The perioperative period is divided into five phases: pre-anesthesia (AWAKE), post-induction (BEGIN), first surgical phase (MIDDLE-1), second surgical phase (MIDDLE-2), and pre-extubation (END). Each patient will undergo both VCV and PCV-VG ventilation modes in random sequence, with a 30-minute washout period between modes.

1. **Randomization and Blinding**
2. **Randomization:** A random sequence will be generated using SAS software. Patients will be assigned to either the VCV-to-PCV-VG group or the PCV-VG-to-VCV group based on allocation order.
3. **Blinding:** This is an open-label study due to the need for real-time adjustment of ventilation by anesthesiologists. However, data analysis will be performed by an independent evaluator, and both patients and postoperative assessors will be blinded to group assignments.
4. **Anesthesia Protocol**

Patients will fast for 8 hours preoperatively. Upon entering the operating room, standard monitoring will be initiated (ECG, SpO₂, NIBP), and baseline vitals recorded. An epidural catheter will be placed at the L1-L2 interspace under local anesthesia. An EIT belt will be placed at the 4th–5th intercostal space, followed by radial arterial and central venous catheterization. All patients will receive 100% oxygen pre-oxygenation.
Anesthesia will be induced with remimazolam (0.3 mg/kg), sufentanil (0.3 µg/kg), cisatracurium (0.3 mg/kg), and propofol (2 mg/kg), followed by tracheal intubation and initiation of mechanical ventilation. Anesthesia will be maintained using combined intravenous and inhalational anesthesia, with intermittent epidural administration of 0.3% ropivacaine and sevoflurane inhalation. BIS will be maintained between 40–60. Fluid therapy and vasoactive drug use will be determined by the attending anesthesiologist to maintain hemodynamic stability. Intra-abdominal pressure during pneumoperitoneum will be set at 12 mmHg. At the end of surgery, anesthetics will be adjusted or withdrawn, and a standard epidural patient-controlled analgesia (PCA) pump will be connected. Patients will be transferred to the PACU after the recovery of spontaneous breathing.

1. **Ventilation Modes for EIT Monitoring**

EIT-1000 (Suzhou Jiantong Medical Technology, China) will be used for monitoring, with a recording frequency of 20 fps. All patients will have PEEP set to 5 cmH₂O, tidal volume to 7 mL/kg, I:E ratio at 1:2, FiO₂ maintained between 30% - 50%, and P_ET_CO₂ controlled within 35 ± 5 mmHg.
 In the VCV-to-PCV-VG group, patients will initially receive volume-controlled ventilation (VCV), followed by a switch to pressure-controlled volume guaranteed (PCV-VG) mode. In the PCV-VG-to-VCV group, the sequence is reversed.

1. **Outcome Measures**

(1) **Primary outcomes:**

- Electrical Impedance Tomography (EIT) parameters: center of ventilation (CoV), global inhomogeneity index (GI), and regional ventilation delay index (RVDI);
- Respiratory mechanics: plateau pressure (Pplat), driving pressure (∆P);
- Oxygenation index (PaO₂/FiO₂).

(2) **Secondary outcomes:**

- Hemodynamic parameters: mean arterial pressure (MAP), heart rate (HR), arterial carbon dioxide (PaCO₂);
- Regional EIT ventilation distribution (ROI 1–4);
- Dynamic lung compliance (Cdyn) during surgery.

1. **Statistical Analysis**

Data will be analyzed using SPSS 26.0. Continuous variables with normal distribution will be expressed as mean ± standard deviation and analyzed using paired t-tests. Non-normally distributed data will be presented as median (interquartile range) and analyzed using Wilcoxon signed-rank tests. Categorical variables will be compared using chi-square or Fisher’s exact tests. A p-value < 0.05 will be considered statistically significant.
