# Supplementary material for: Evaluation of mechanical ventilation modes in the laparoscopic perioperative period with electrical impedance tomography
Source: PLoS One. 2025 Sep 8;20(9):e0331194. doi: 10.1371/journal.pone.0331194 (PMC12416645; doi:10.1371/journal.pone.0331194)
Supplement: S3 File — (DOCX) [file pone.0331194.s003.docx]

Power Calculation

**We referred to the study by Guttmann et al. (Intensive Care Medicine, 1997), which compared volume-controlled ventilation (VCV) and pressure-controlled ventilation (PCV) in an experimental lung injury model**^[1]^. In their results, the average difference in dynamic compliance (Cdyn) between the two modes was reported as approximately 8.0 mL/cmH₂O, with a standard deviation of 13.0 mL/cmH₂O. Using these values, we calculated the effect size as:

$$\text{Cohe}\text{n}'\text{s d = }\frac{8.0}{13.0}\approx0.615$$

Assuming a **two-tailed *α* = 0.05** ($Z_{1-\alpha/2}\approx1.96$), a **power of 80% (*β* = 0.2,** $Z_{1-\beta}\approx0.84$**)**, and a **paired design** suitable for our crossover trial, the required number of participants was calculated as:

$$n=\left(\frac{Z_{1-\alpha/2}+Z_{1-\beta}}{d}\right)^{2}=\left(\frac{1.96+0.84}{0.615}\right)^{2}\approx(4.55)^{2}=20.7\approx21$$

To accommodate patients who would be excluded based on predefined criteria, we adjusted the sample size accordingly (estimated at 20%):

$$n_{\text{adjusted }}=\frac{n}{1-\text{ exclusion rate }}=\frac{21}{1-0.20}=\frac{21}{0.8}=26.25$$

This means that **at least 27 participants** would need to complete the study with usable data to achieve the desired statistical power. Given the randomized crossover design, in which each subject undergoes both ventilation modes at different time points, we doubled the adjusted estimate to ensure balanced representation and within-subject comparison across conditions:

$$n_{total}=n_{\text{adjusted }}\times2=27\times2=54$$

To provide further margin for unexpected exclusions and variability, a total of 61 participants were ultimately enrolled, exceeding the calculated requirement and ensuring robust statistical power for detecting moderate physiologic differences.

[1] Guttmann J, Eberhard L, Fabry B, Bertschmann W, Wolff G. Comparison of volume-controlled and pressure-controlled ventilation: effects on lung mechanics and gas exchange in pigs with oleic acid-induced lung injury. Intensive Care Med. 1997;23(5):472–478. doi:10.1007/s001340050352.
